# Endothelial Cell-Derived SO_2_ Controls Endothelial Cell Inflammation, Smooth Muscle Cell Proliferation, and Collagen Synthesis to Inhibit Hypoxic Pulmonary Vascular Remodelling

**DOI:** 10.1155/2021/5577634

**Published:** 2021-04-17

**Authors:** Xin Liu, Shangyue Zhang, Xiuli Wang, Yuanyuan Wang, Jingyuan Song, Chufan Sun, Guozhen Chen, Guosheng Yang, Yinghong Tao, Yongyan Hu, Dingfang Bu, Yaqian Huang, Junbao Du, Hongfang Jin

**Affiliations:** ^1^Department of Pediatrics, Peking University First Hospital, Beijing 100034, China; ^2^Department of Pediatrics, The Affiliated Yantai Yuhuangding Hospital of Qingdao University, Yantai 264000, China; ^3^Laboratory Animal Facility, Peking University First Hospital, Beijing 100034, China; ^4^Central Laboratory, Peking University First Hospital, Beijing 100034, China; ^5^Key Laboratory of Molecular Cardiology, Ministry of Education, Beijing 100091, China

## Abstract

Hypoxic pulmonary vascular remodelling (PVR) is the major pathological basis of aging-related chronic obstructive pulmonary disease and obstructive sleep apnea syndrome. The pulmonary artery endothelial cell (PAEC) inflammation, and pulmonary artery smooth muscle cell (PASMC) proliferation, hypertrophy and collagen remodelling are the important pathophysiological components of PVR. Endogenous sulfur dioxide (SO_2_) was found to be a novel gasotransmitter in the cardiovascular system with its unique biological properties. The study was aimed to investigate the role of endothelial cell- (EC-) derived SO_2_ in the progression of PAEC inflammation, PASMC proliferation, hypertrophy and collagen remodelling in PVR and the possible mechanisms. EC-specific aspartic aminotransferase 1 transgenic (EC-AAT1-Tg) mice were constructed *in vivo*. Pulmonary hypertension was induced by hypoxia. Right heart catheterization and echocardiography were used to detect mouse hemodynamic changes. Pathologic analysis was performed in the pulmonary arteries. High-performance liquid chromatography was employed to detect the SO_2_ content. Human PAECs (HPAECs) with lentiviruses containing AAT1 cDNA or shRNA and cocultured human PASMCs (HPASMCs) were applied *in vitro*. SO_2_ probe and enzyme-linked immunosorbent assay were used to detect the SO_2_ content and determine p50 activity, respectively. Hypoxia caused a significant reduction in SO_2_ content in the mouse lung and HPAECs and increases in right ventricular systolic pressure, pulmonary artery wall thickness, muscularization, and the expression of PAEC ICAM-1 and MCP-1 and of PASMC Ki-67, collagen I, and *α*-SMA (*p* < 0.05). However, EC-AAT1-Tg with sufficient SO_2_ content prevented the above increases induced by hypoxia (*p* < 0.05). Mechanistically, EC-derived SO_2_ deficiency promoted HPAEC ICAM-1 and MCP-1 and the cocultured HPASMC Ki-67 and collagen I expression, which was abolished by andrographolide, an inhibitor of p50 (*p* < 0.05). Meanwhile, EC-derived SO_2_ deficiency increased the expression of cocultured HPASMC *α*-SMA (*p* < 0.05). Taken together, these findings revealed that EC-derived SO_2_ inhibited p50 activation to control PAEC inflammation in an autocrine manner and PASMC proliferation, hypertrophy, and collagen synthesis in a paracrine manner, thereby inhibiting hypoxic PVR.

## 1. Introduction

Aging is an important risk factor for a variety of diseases [1–3]. With the increase of the global elderly population, the incidence of aging and age-related diseases such as chronic obstructive pulmonary disease (COPD) and obstructive sleep apnea syndrome (OSAS) has also been gradually increased [4, 5]. Among them, hypoxic pulmonary hypertension (PH) and pulmonary vascular remodelling (PVR) are the critical pathological basis. PVR includes pulmonary artery endothelial cell (PAEC) dysfunction, and pulmonary artery smooth muscle cell (PASMC) proliferation, hypertrophy, and collagen accumulation [6–9]. Previous studies reported that an imbalance among small vasoactive molecules played a critical role in the progression of PVR. The imbalance among protein-derived bioactive molecules, active lipid mediators, small nucleic acids, and gaseous signalling molecules is predominantly involved in the pulmonary artery structural changes and the abnormal vasoconstriction and vasorelaxation [10–16]. However, the mechanisms underlying the excessive PAEC inflammation, PASMC proliferation, hypertrophy, and collagen remodelling remain unclear.

Recent studies have shown that endothelial cells (ECs) play a critical role in maintaining vascular homeostasis, while EC dysfunction leads to various vascular diseases [17, 18]. For example, Xue et al. observed that the overexpression of EC-derived cyclophilin A caused spontaneous PH by promoting PAEC inflammation and PASMC proliferation [19]. Moreover, EC dysfunction and endothelial-to-mesenchymal cell transition enhanced collagen accumulation in the pathogenesis of human fibrotic diseases [20]. These results suggest that ECs might affect the function of ECs and other neighboring cells (primarily smooth muscle cells [SMCs]) in the vascular walls in an autocrine/paracrine manner, by which ECs play an important role in the development of PVR. However, the mechanisms by which ECs affect the behavior of PAECs and PASMCs to play a role in the development of PVR are complex and have not yet been fully elucidated.

In 2008, the endogenous sulfur dioxide (SO_2_)/aspartate aminotransferase (AAT) pathway was found to exist in the vascular ECs of rats [21]. More recently, the production of endogenous SO_2_ catalyzed by AAT1 has been identified in human PAECs (HPAECs) [22]. As a gasotransmitter, SO_2_ has a series of important advantages, including sustained production, rapid diffusion, and free passage through cell membranes with a short half-life. It exerts a variety of biological functions in the cardiovascular system. For example, *in vivo*, the exogenous SO_2_ donor showed a protective role in rat atherosclerosis and sepsis-induced cardiac dysfunction via the inhibition of cellular inflammation [23, 24]. *In vitro*, the deficiency of SO_2_ contributed to cellular proliferation and collagen accumulation [25]. Moreover, the deficiency of endogenous SO_2_ might be implicated in the pathogenesis of myocardial hypertrophy [26]. These data strongly suggest that EC-derived SO_2_ might play a regulatory role in PAEC inflammation in an autocrine manner and in PASMC proliferation, hypertrophy, and collagen deposition in a paracrine manner. As such, in the present study, we further explored whether EC-derived SO_2_ might control PAEC inflammation in an autocrine manner and control PASMC proliferation, hypertrophy, and collagen deposition in a paracrine manner to reveal a new mechanism for PAEC inflammation and PAEC-PASMC communication in the control of PASMC proliferation, hypertrophy, and collagen remodelling by SO_2_.

Recent data have suggested that EC inflammation, SMC proliferation, and collagen synthesis are under the control of nuclear factor-*κ*B (NF-*κ*B) [27, 28]. Briefly, some stimuli, such as tumor necrosis factor *α* (TNF-*α*), lead to the activation of the I*κ*B kinase complex, allowing I*κ*B to undergo phosphorylation and degradation, and NF-*κ*B dimers (thought to be p50 heterodimers primarily) to translocate to the nucleus to promote gene transcription [29]. The inhibition of p50 evidently restricted E-selectin expression induced by TNF-*α* in human umbilical vein ECs, and NF-*κ*B activation triggered bladder SMC collagen biosynthesis and proliferation [30, 31]. But the regulatory mechanisms for p50 are unclear so far. Intriguingly, endogenous SO_2_ exhibited a protective effect by repressing NF-*κ*B activation in adipocytes and macrophages.

Based on these evidences, in this study, we aimed to determine the possible role of EC-derived SO_2_ in the progression of hypoxic PVR and its possible underlying mechanisms in association with the PAEC inflammatory response, PASMC proliferation, hypertrophy, and collagen production to reveal a new mechanism for PAEC inflammation and PAEC-PASMC communication in the control of PASMC proliferation, hypertrophy, and collagen remodelling by SO_2_.

## 2. Materials and Methods

### 2.1. Animal Model

Endothelial cell-specific AAT1 transgenic (EC-AAT1-Tg) and wild-type (WT) littermate mice (C57BL/6J background) were purchased from Cyagen Biosciences (Suzhou, China). Genotype was confirmed using polymerase chain reaction analysis in 10-day-old mice. Male mice aged 10–12 weeks were randomly divided into WT, hypoxic WT (WT+H), EC-AAT1-Tg, and hypoxic EC-AAT1-Tg (EC-AAT1-Tg+H) groups. The mice of the WT and EC-AAT1-Tg groups breathed in room air (21% O_2_), whereas hypoxic mice were placed in a small animal hypoxic chamber (9 ± 0.5% O_2_) (XBS-02B; Hangzhou Aipu Instrument Co., Ltd., China) 8 h daily for 5 weeks to induce hypoxic PH [32]. All institutional and national guidelines for the care and use of animals (fisheries) were followed. The animal experiment was approved by the Laboratory Animal Ethics Committee of Peking University First Hospital (Ethics No. 201526).

### 2.2. High-Performance Liquid Chromatography (HPLC) Quantitative Detection of SO_2_ Content in Mouse Lung Tissue

As mentioned earlier, HPLC (Agilent 1200 series; Agilent Technologies, CA, USA) was used to detect the SO_2_ content in mouse lung tissue. Briefly, 100 *μ*L of the standard sodium sulfite and a sample of mouse lung tissue homogenate were mixed with 70 *μ*L of sodium borohydride (0.212 mol/L) in Tris-HCl (0.05 mol/L, pH 8.5). The mixture was then incubated at room temperature for 30 min. Next, 5 *μ*L of monobromobimane (70 mmol/L) in acetonitrile was mixed with the 170 *μ*L mixture. After the above mixture was incubated at 42°C for 10 min, 40 *μ*L of perchloric acid (1.5 mol/L) was added. To remove the protein precipitates and neutralize the mixture, the mixture was centrifuged (12,400 g) at 25°C for 10 min, and 10 *μ*L of Tris-HCl (2 mol/L, pH 3.0) was added to the supernatant. Finally, the HPLC operation and result analysis were performed as described previously [33].

### 2.3. Detection of Mouse Right Ventricular Systolic Pressure (RVSP) Changes by Right Heart Catheterization

The RVSP of mice was directly measured by catheterization. The mice were anesthetized by intraperitoneal injection of 0.5% sodium pentobarbital (0.1 ml/10 g). A catheter was inserted through the external jugular vein to reach the right ventricle of the mouse. The BL-420F Biological Function Experimental System (Chengdu Taimeng Instrument Co. Ltd., China) was used to measure the mouse RVSP to predict the changes in pulmonary artery pressure [32].

### 2.4. Measurement of Mouse Pulmonary Acceleration Time (PAT) and Pulmonary Ejection Time (PET) Using Echocardiography

Echocardiography was performed using the Vevo 2100 high-resolution imaging system (Visualonics, Toronto, Canada). The mice were initially anesthetized by intraperitoneal injection of 0.5% sodium pentobarbital (0.1 ml/10 g). Pulsed-wave Doppler recording of the pulmonary blood flow was obtained from the parasternal short axis view at the aortic valve level. Samples were positioned at the tip of the pulmonary valve leaflets and aligned to maximize laminar flow as described previously. PAT (defined as the time from the onset of flow to peak velocity by pulsed-wave Doppler recording) and PET (the time from the onset to the termination of pulmonary flow) variables were determined. All measured variables were the average of 3–5 cardiac cycles [34].

### 2.5. Preparation and Morphological Analysis of Mouse Lung Tissue

The mice were killed by cervical dislocation. Their lungs were removed and rinsed with 4°C phosphate-buffered saline (PBS). The lungs were then fixed in 4% paraformaldehyde solution for 24 h to prepare paraffin-embedded sections or frozen sections of lung tissue. The sections were stained with hematoxylin and eosin (HE) and Hart's method for morphological analysis. As previously reported [35], the degree of muscularization can be determined by the ratio of the number of nonmuscular vessels (NMV), partial muscular arteries (PMA), and muscular arteries (MA). PVR can be quantitatively analyzed by calculating the percentage of 15–50 *μ*m pulmonary arteriole muscularization.

### 2.6. Immunofluorescence

Frozen sections of lung tissue and cells were rinsed with PBS and fixed with 4% paraformaldehyde solution for 20 min. Tissue sections and cells were blocked with bovine serum albumin and incubated with the primary antibodies at 4°C overnight (vWF: 1 : 200, Zhongshan Jinqiao Biotechnology Co., Ltd., China; AAT1 1 : 50, Sigma-Aldrich, USA; p-p50 1 : 50 and ICAM-1 1 : 20, Santa Cruz Biotechnology Company, USA; p50 1 : 100 and Ki-67 1 : 100, Cell Signaling Technology Company USA; F4/80 1 : 300, *α*-SMA 1 : 300, collagen 1 : 25 and MCP-1 1 : 100, Abcam Company, UK). On the second day, the secondary fluorescent antibodies were incubated in the dark. All sections were counterstained with nuclear 4,6-diamino-2-phenylindole (DAPI; Zhongshan Jinqiao Biotechnology Co., Ltd., China). Immunofluorescence imaging was performed using a confocal laser scanning microscope (Leica, Wetzlar, Germany) for observation and comparison.

### 2.7. Primary HPAEC Culture

Primary HPAECs and primary EC culture system were purchased from PriCells Biomedical Technology Co., Ltd. (Wuhan, China); cells at 4-6 generation were used for experiments. HPAECs were transfected with lentivirus containing AAT1 shRNA or cDNA (Cyagen Biosciences, Suzhou, China) for 2–3 days to induce AAT1 knockdown or overexpression. 1% O_2_ was used to induce a hypoxic cell inflammation model.

### 2.8. Coculture of Primary Human PASMCs (HPASMCs) and HPAECs

Primary HPASMCs and primary SMC culture system were purchased from Lifeline Cell Technology (USA). Transwell plates (Corning, China) were used for the coculture of HPASMCs and HPAECs. We seeded HPASMCs in the lower compartment of the transwell plates by using the SMC culture system. Infected HPAECs were planted in the upper compartment with an EC culture system and were separated from the lower compartments by a microporous membrane. An inhibitor of p50 andrographolide (4 *μ*M, Andro, Selleck, USA) was used for experiments [30].

### 2.9. SO_2_ Probe-Based *In Situ* Detection of SO_2_ Content in HPAECs

The SO_2_ fluorescent probe was used to analyze the SO_2_ content in HPAECs *in situ* as previously reported [22]. After the unbound SO_2_ probe was removed and the nuclei were stained with DAPI, we observed the red fluorescence intensity of the SO_2_ probe under a high-resolution confocal laser microscope.

### 2.10. Western Blot

Lung tissues or cells were lysed in lysis buffer to obtain total protein. Denatured proteins were separated by 10% sodium dodecyl sulfate-polyacrylamide gel electrophoresis and transferred to nitrocellulose membranes (Amerisco, USA). The membranes were incubated with specific primary antibodies (GAPDH 1 : 4000, *β*-actin 1 : 4000, AAT1 1 : 1000, ICAM-1 1 : 400, MCP-1 : 1000, Ki-67 1 : 500, collagen I 1 : 1000, *α*-SMA 1 : 1000 and p-p50 1 : 1000) and secondary antibodies (1 : 2000) conjugated with horseradish peroxidase. The FluorChem M MultiFluor System (Proteinsimple, USA) was used to scan the protein bands in grayscale. The ImageJ software was used to quantitatively analyze each band, and each protein band was corrected with its own internal reference GAPDH or *β*-actin grayscale [33].

### 2.11. p50 DNA-Binding Activity Detected by Active Motif-Enzyme-Linked Immunosorbent Assay (ELISA)

Active motif-ELISA (Active Motif, USA) was used to determine the DNA-binding activity of p50 in HPAEC nuclear protein. The nuclear protein extraction steps were initially performed as described earlier [14]. The DNA-binding activity of p50 was then evaluated according to manufacturer's instructions. In short, the binding buffer, the sample diluted with lysate, 1x washing buffer, p50 primary antibody (1 : 1000, prepared with 1x antibody binding buffer), secondary antibody, and color developing solution were added in turn. We observed the color change during the experiment. The experiment was stopped when the color turned to moderate dark blue by adding stop solution, and then, the color turned yellow. Finally, we measured the absorbance value at 450 nm as soon as possible.

### 2.12. Adhesion Test of THP-1 Monocyte Cell Line and HPAECs

After inducing the HPAEC inflammatory response, THP-1 mononuclear cells initially stained with the red fluorescent Dil (Beyotime Biotechnology, China) were added to the HPAECs. HPAECs and THP-1 cells were incubated at 37°C for 1 h. PBS was then used to remove unadhered THP-1 cells. Subsequently, we fixed the cells with 4% paraformaldehyde solution for 20 min. Finally, the mounting medium containing DAPI was added, and fluorescence was observed under an immunofluorescence microscope.

### 2.13. Data Analysis

Statistical analyses were performed with the SPSS 21.0 software (SPSS Inc., USA). Data were expressed as mean ± SEM and analyzed using one-way ANOVA. *p* values < 0.05 were considered statistically significant.

## 3. Results

### 3.1. Hypoxia Caused Reduction in EC-Derived SO_2_

**Figure 1:**
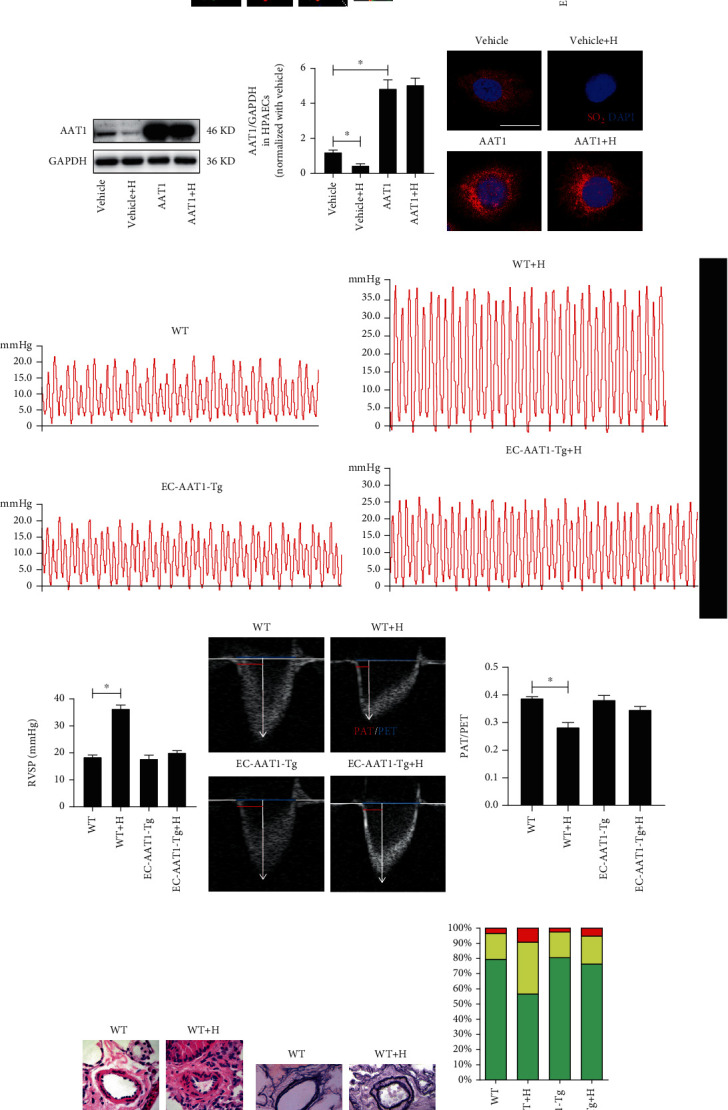
Increased EC-derived SO_2_ ameliorates hypoxia-induced PH. (a) Immunofluorescence *in situ* detection of AAT1 protein expression in mouse PAECs. Green fluorescence represents AAT1 protein, and red fluorescence represents vWF, a marker for ECs; the nuclei were stained with DAPI. (b) HPLC method was performed to detect SO_2_ content in mouse lung tissue (*n* = 5 − 17). (c) Western blot analysis was carried out to detect the expression level of AAT1 protein in HPAECs (*n* = 10). (d) Red SO_2_ fluorescent probe method was used to determine the SO_2_ level in HPAECs, and the nuclei of HPAECs were stained with DAPI. (e, f) Catheter method was performed to detect mouse right ventricular systolic pressure (RVSP) (*n* = 6 − 8). (g, h) The ratio of pulmonary acceleration time (PAT) to pulmonary ejection time (PET) of the mouse pulmonary artery blood flow was measured using ultrasound (*n* = 8). (i, j) HE and Hart's methods were used to detect PVR in mice. (k) Hart's method was conducted to analyze the degree of muscularization of small pulmonary vessels in mice (*n* = 8); NMV: nonmuscular vessels; PMA: partial muscular arteries; MA: muscular arteries. The data were expressed as mean ± SEM, ^∗^*p* < 0.05, scale bar: 20 *μ*m.

Immunofluorescence and HPLC showed decreased AAT1 protein expression in PAECs and SO_2_ content in the lungs of WT mice under hypoxia compared with control WT mice (Figures 1(a) and 1(b)) (*p* < 0.05). By Western blot analysis and SO_2_ fluorescent probe method, we further confirmed that compared with the control vehicle group, the levels of AAT1 protein and SO_2_ were downregulated in hypoxic HPAECs, whereas overexpression of AAT1 significantly improved the endogenous SO_2_ level in ECs and prevented the decrease in EC-derived SO_2_ caused by hypoxia (Figures 1(c) and 1(d)) (*p* < 0.05).

### 3.2. Increased EC-Derived SO_2_ Ameliorated Hypoxia-Induced PVR and PH *In Vivo*

To reveal the role of EC-derived SO_2_ in the pathogenesis of hypoxic PVR, we constructed EC-AAT1-Tg mice to increase SO_2_ levels in mouse PAECs (Figure S1a). We found an increased expression of AAT1 protein in PAECs and SO_2_ content in the lung of EC-AAT1-Tg mice, compared with the mice in the WT group (Figures 1(a) and 1(b)) (*p* < 0.05). After exposure to intermittent hypoxia for 5 weeks, both the RVSP detected by the right heart catheter and the PAT/PET ratio in this experiment suggested that hypoxic WT (WT+H) mice developed significant PH compared with the control WT mice. We also observed that markers of PVR, the thickness of pulmonary artery walls, and the proportion of muscularized arteries were dramatically enhanced in mice of the WT+H group by HE and Hart's methods. However, compared with mice in EC-AAT1-Tg group with increased EC-SO_2_ content, hypoxia did not induce significant PVR and PH in mice of EC-AAT1-Tg+H group (Figures 1(e)–1(k)) (*p* < 0.05). The above results suggested that the decrease in SO_2_ in ECs resulted in increased vascular remodelling and hypoxic PH in mice.

### 3.3. Increased EC-Derived SO_2_ Ameliorated Hypoxia-Induced PAEC Inflammation, PASMC Proliferation, Hypertrophy, and Collagen Production

**Figure 2:**
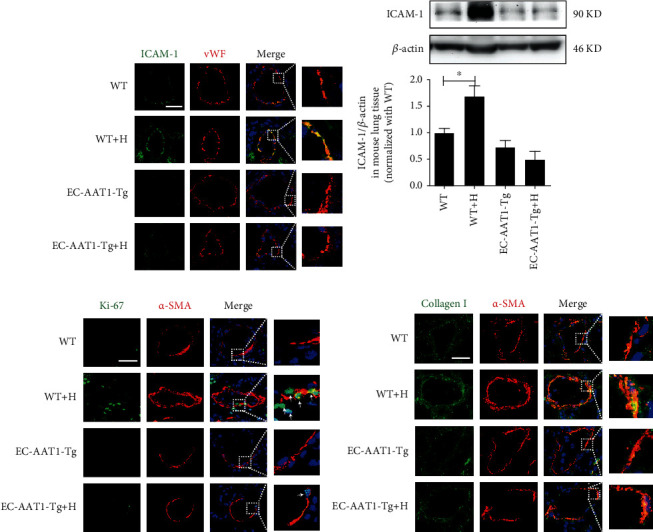
Increased EC-derived SO_2_ ameliorates hypoxia-induced PAEC inflammation, PASMC proliferation, hypertrophy, and collagen production *in vivo*. (a) Immunofluorescence *in situ* detection of ICAM-1 protein expression in mouse PAECs; green fluorescence represents ICAM-1 protein. (b) Western blot analysis was performed to detect the expression level of ICAM-1 protein in mouse lung tissue (*n* = 10). (c, d) Immunofluorescence *in situ* detection of Ki-67, *α*-SMA, and collagen I protein expression in mouse PASMCs; green fluorescence represents Ki-67 and collagen I protein, and red fluorescence represents *α*-SMA. The data were expressed as mean ± SEM, ^∗^*p* < 0.05, scale bar: 20 *μ*m.

We next sought to determine the role of EC-derived SO_2_ in PAEC inflammatory reaction, PASMC proliferation, hypertrophy, and collagen synthesis, which dramatically contribute to PVR. *In vivo*, by immunofluorescence *in situ* detection of ICAM-1 and MCP-1 expression and macrophage infiltration in pulmonary vasculature, we found that compared with WT mice, hypoxia dramatically increased the expression of ICAM-1 and MCP-1, known markers of EC inflammatory process in mouse PAECs, and macrophage infiltration in the pulmonary arteries of WT+H mice. Moreover, Western blot quantitative determination of ICAM-1 protein in mouse lung showed similar results (Figures 2(a) and 2(b), Figure S2a and S2b) (*p* < 0.05). But the treatment with EC-AAT1-Tg to increase SO_2_ level successfully repressed hypoxia-induced increases in the protein expression of ICAM-1 and MCP-1, and macrophage infiltration in mouse lung tissue (Figures 2(a) and 2(b), Figure S2a and S2b) (*p* < 0.05). Consistent with the *in vivo* results, we found that AAT1 overexpression to increase the content of EC-derived SO_2_ evidently inhibited hypoxia-induced increases in ICAM-1 and MCP-1 in HPAECs and THP-1 cell adhesion to HPAECs *in vitro* (Figure S2c-S2f) (*p* < 0.05). These data suggested that the increase in SO_2_ in ECs could inhibit hypoxic vascular inflammation in an autocrine manner.

We then evaluated the effect of EC-derived SO_2_ on hypoxic PASMC proliferation, hypertrophy, and collagen deposition *in vivo*. Compared with the mice in the WT group, immunofluorescence showed enhanced Ki-67 staining in the pulmonary arteries of the WT+H group. While compared with the mice of EC-AAT1-Tg group, no significant increase was found in the expression of Ki-67 induced by hypoxia in the mice of EC-AAT1-Tg+H group (Figure 2(c)). In addition, immunofluorescence showed an increased *α*-SMA expression, a marker of SMC hypertrophy, in the pulmonary arteries of WT mice under hypoxia compared with WT mice under normoxia, while there was no significant difference in *α*-SMA expression between EC-AAT1-Tg mice with and without hypoxic exposure (Figures 2(c) and 2(d)). We also observed that the increased collagen I in PASMCs of mice under hypoxia was successfully repressed in EC-AAT1-Tg mice with sufficient SO_2_ (Figure 2(d)). The above results indicated that increased EC-derived SO_2_ ameliorated hypoxia-induced PASMC proliferation, hypertrophy, and collagen accumulation in a paracrine manner.

### 3.4. EC-Derived SO_2_ Deficiency Activated HPAEC Inflammation, HPASMC Proliferation, Hypertrophy and Collagen Synthesis *In Vitro*

**Figure 3:**
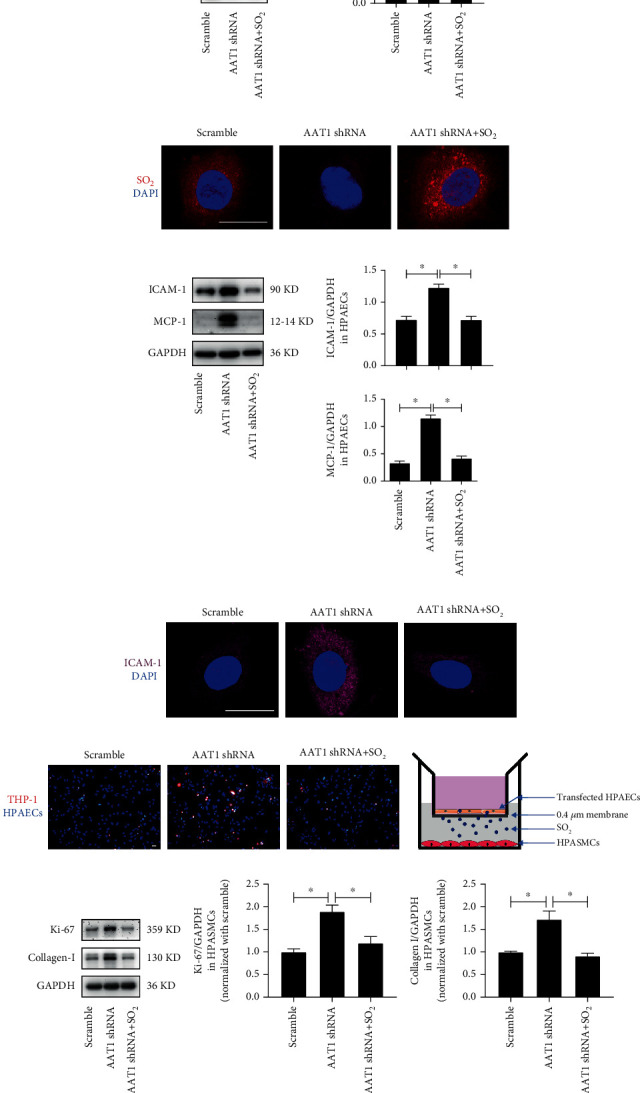
EC-derived SO_2_ deficiency activates HPAEC inflammation, HPASMC proliferation, and collagen synthesis *in vitro*. (a) Western blot analysis was performed to detect the expression level of AAT1 protein in HPAECs (*n* = 12). (b) The red SO_2_ fluorescent probe method was used to detect the SO_2_ level in HPAECs; the nuclei were stained with DAPI. (c) Western blot analysis was used to detect the expression level of ICAM-1 and MCP-1 protein in HPAECs (*n* = 12). (d) Immunofluorescence method was used to detect the expression level of ICAM-1 protein in HPAECs *in situ*. Purple fluorescence represents ICAM-1 protein. (e) Fluorescence method was conducted to detect the adhesion of THP-1 cells and HPAECs. The red Dil color marks the THP-1 cells, and the DAPI color marks the nuclei of HPAECs. (f) Diagram of the transwell used for the coculture system *in vitro*. (g) Western blot analysis method to detect the expression level of Ki-67 and collagen I protein in HPASMCs cocultured with HPAECs (*n* = 10). The data were expressed as mean ± SEM, ^∗^*p* < 0.05, scale bar: 20 *μ*m.

To directly identify the role of EC-derived SO_2_ in HPAEC inflammatory process, HPASMC proliferation, hypertrophy, and collagen production, we infected HPAECs with lentivirus containing AAT1 shRNA to reduce the expression of the key enzyme AAT1 for SO_2_ production. By Western blot analysis and the SO_2_ fluorescent probe method, we confirmed that the AAT1 protein level and SO_2_ probe staining significantly decreased in AAT1-knockdown (AAT1 shRNA) HPAECs. However, treatment with SO_2_ donor restored SO_2_ level in HPAECs (Figures 3(a) and 3(b)) (*p* < 0.05).

Compared with the scramble control group, we further verified that the protein expression of ICAM-1 and MCP-1 and the number of monocyte adhesion to HPAECs in the AAT1 shRNA group all evidently increased. However, supplementation with SO_2_ donor successfully inhibited these increases in AAT1 shRNA HPAECs (Figures 3(c)–3(e)) (*p* < 0.05). In addition, HPASMCs cocultured with HPAECs of the AAT1 shRNA group showed increased expression of Ki-67, collagen I, and *α*-SMA, which were also suppressed by supplementation with SO_2_ donor in HPAECs (Figures 3(f) and 3(g), Figure S3a and S3b) (*p* < 0.05). The above studies directly suggested that SO_2_ derived from ECs regulated the function of HPAECs and HPASMCs. Moreover, the deficiency of EC-derived SO_2_ activated the inflammatory reaction of HPAECs in an autocrine manner, and the proliferation, collagen biosynthesis, and hypertrophy of HPASMCs in a paracrine manner.

### 3.5. EC-Derived SO_2_ Inhibited p50 Activation to Repress PAEC Inflammation, and PASMC Proliferation, and Collagen Deposition

**Figure 4:**
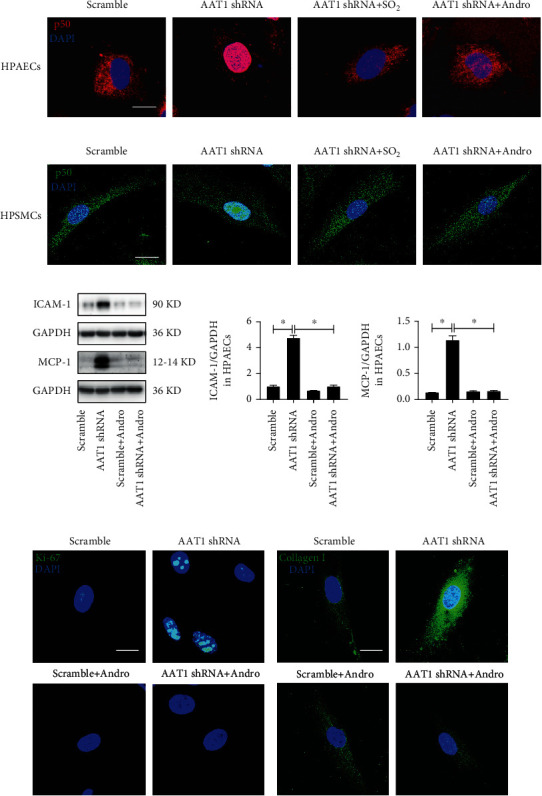
EC-derived SO_2_ inhibits p50 activation to repress PAEC inflammation, PASMC proliferation, and collagen deposition. (a) Immunofluorescence *in situ* detection of p50 protein distribution in HPAECs. Red fluorescence represents p50 protein, and DAPI staining labels HPAEC nuclei. (b) Immunofluorescence *in situ* detection of p50 protein distribution in HPASMCs cocultured with HPAECs. Green fluorescence represents p50 protein, and DAPI staining labels HPASMC nuclei. (c) Western blot analysis was used to detect the expression level of ICAM-1 and MCP-1 protein in HPAECs (*n* = 12). (d, e) Immunofluorescence *in situ* detection of Ki-67 and collagen I protein expression in HPASMCs cocultured with HPAECs. Green fluorescence represents Ki-67 and collagen I protein. Andro: andrographolide, an inhibitor of p50. Data were expressed as mean ± SEM, ^∗^*p* < 0.05, scale: 20 *μ*m.

Considering that NF-*κ*B (particularly p50 heterodimers) plays a critical role in cell inflammation, proliferation, and collagen metabolism, we further observed p50 activation in HPAECs and HPASMCs to reveal the mechanism by which EC-derived SO_2_ played a protective effect. The nuclear translocation of p50 was significantly enhanced in HPAECs of the AAT1 shRNA group compared with the scramble group, but this effect was reversed by the treatment with SO_2_ donor (Figure 4(a)).

Furthermore, in the hypoxia-induced HPAEC inflammatory response, the phosphorylation, nuclear translocation, and activity of p50 evidently increased in HPAECs of the hypoxic vehicle (vehicle+H) group compared with the normoxic vehicle group. However, no significant differences were found in AAT1-overexpressed HPAECs between the normoxic and hypoxic treatment groups (Figure S4a-S4c) (*p* < 0.05). Moreover, the *in vivo* EC-AAT1-Tg abolished hypoxia-induced p50 activation (Figure S4d).

Meanwhile, we found that with inhibition of endogenous SO_2_ generation in HPAECs, nuclear translocation of p50 in HPASMCs cocultured with HPAECs was activated significantly, which was repressed by the treatment with SO_2_ donor as well (Figure 4(b)). These results suggested that p50 may be the molecular target of SO_2_.

To investigate whether the inflammatory reaction of HPAECs and the proliferation and collagen accumulation of HPASMCs were mediated by p50, intervention with Andro, an inhibitor of p50, demonstrated that Andro successfully prevented nuclear translocation of p50 induced by the decrease in EC-derived SO_2_ in HPAECs and HPASMCs (Figures 4(a) and 4(b)). Meanwhile, the enhanced expression levels of ICAM-1 and MCP-1 in HPAECs of the AAT1shRNA group were significantly inhibited by Andro treatment (Figure 4(c)) (*p* < 0.05). In agreement with the results of HPAECs, the increased expression of Ki-67 and collagen I in HPASMCs cocultured with HPAECs of the AAT1shRNA group was also inhibited by Andro (Figures 4(d) and 4(e)). The above results indicated that Andro completely blocked the augmentation of HPAEC inflammation, HPASMC proliferation, and collagen production caused by the decrease in EC-derived SO_2_ and p50 was a key molecular target for the control of PAEC inflammation, PASMC proliferation, and collagen remodelling by SO_2_.

## 4. Discussion

We demonstrated that EC-derived SO_2_ was an important endogenous controller of hypoxic PVR. It inhibited PAEC inflammatory process in an autocrine manner and PASMC proliferation, hypertrophy, and collagen accumulation in a paracrine manner. NF-*κ*B p50 signalling might mediate the above effect of EC-derived SO_2_.

PVR is the main pathological feature of aging-related COPD and OSAS [6, 7]. The key roles of PAEC inflammation, and PASMC proliferation, hypertrophy, and collagen deposition in the pathogenesis of PVR have been suggested by previous studies [8, 9, 11, 25, 36]. However, the mechanisms underlying the endogenous control of PVR have not been identified. Evidence supports that ECs play an important role in the pathogenesis of PVR [18, 19]. However, how PAEC inflammation is regulated and whether PASMC proliferation, hypertrophy, and collagen synthesis are controlled through PAEC-PASMC communication in PVR are not completely clear.

Recently, the gasotransmitter SO_2_ with a range of properties, including sustained production, rapid diffusion, and broad functions has been shown to be produced endogenously in HPAECs [21, 22]. Previously, our laboratory and others showed the critical roles of SO_2_ donor in controlling cellular collagen metabolism, inflammation, proliferation, and hypertrophy in the pathogenesis of cardiovascular diseases [23–26]. In the present study, we found that the decrease in endogenous SO_2_ in hypoxic PH mice with significant PVR and HPAECs, and hypoxic exposure caused the downregulated expression of AAT1 protein in mouse PAECs and HPAECs. This finding indicated that the endogenous SO_2_/AAT1 pathway in ECs was probably associated with hypoxic PH and PVR.

To investigate the regulatory effects of EC-derived SO_2_ on the development of hypoxic PH and PVR, and the mechanisms, EC-AAT1-Tg mice with forced expression of AAT1 in ECs were used in the *in vivo* experiment, and AAT1 cDNA was transfected with a lentivirus vector to hold the AAT1 expression in *in vitro* experiment. Unlike WT mice, EC-AAT1-Tg mice did not respond to hypoxic stimulation, demonstrated by the results that hypoxic exposure induced an increase in RVSP and PVR in WT mice, but normoxic EC-AAT1-Tg mice exhibited similar RVSP and vascular structure to EC-AAT1-Tg mice exposed to hypoxia. Furthermore, hypoxia-induced the expression of inflammatory factor ICAM-1 and MCP-1 in the PAECs and macrophage infiltration in the pulmonary arteries were alleviated in EC-AAT1-Tg mice. In accordance with the results *in vivo*, AAT1 overexpression blocked the hypoxia-stimulated expression of ICAM-1 and MCP-1 in HPAECs and monocyte adhesion to HPAECs. It is known that PVR is mainly produced by a thickening of the lamina media due to an increase in PASMC proliferation, hypertrophy, and collagen deposition [8, 11, 25, 36]. Especially, cell hypertrophy is a well-known cell senescence hallmark [2, 37]. Therefore, we observed the effect of EC-derived SO_2_ on the PASMC behaviors *in vivo*. The data showed that hypoxia stimulated the increased expression of proliferative marker Ki-67, hypertrophy marker *α*-SMA, and classical collagen I in PASMCs in WT mice, but not in EC-AAT1-Tg mice. The abovementioned results suggested an important role of EC-derived SO_2_ in the regulation of hypoxic PVR.

Conversely, to directly investigate the effect of EC-derived SO_2_ on the HPAEC inflammation, and HPASMC proliferation, hypertrophy, and collagen deposition, HPAECs were transfected with human AAT1 shRNA lentivirus to decrease the level of EC-derived SO_2_, and SO_2_ donor was used for rescuing the effect of AAT1 knockdown. As we expected, the deficiency of endogenous SO_2_ in HPAECs caused spontaneous overexpression of ICAM-1 and MCP-1 in ECs and monocyte adhesion to HPAECs. Recently, the insufficiency of endogenous SO_2_ in adipocytes and macrophages has been found to induce spontaneous inflammatory responses, strengthening the protective role of endogenous SO_2_ in the cardiovascular system [38, 39]. Interestingly, HPASMCs cocultured with AAT1 shRNA HPAECs presented a PH phenotype characterized by increased Ki-67 and *α*-SMA expression, and accumulation of collagen I in association with the decreased SO_2_ content in HPAECs. These results further supported that EC-derived SO_2_ was an important endogenous controller of PVR. EC-derived SO_2_ inhibited the PAEC inflammatory process in an autocrine manner, and the coculture of HPAECs and HPASMCs further verified that EC-derived SO_2_ inhibited PASMC proliferation, hypertrophy, and collagen accumulation in a paracrine manner.

To demonstrate the mechanism by which EC-derived SO_2_ controls PAEC inflammation, PASMC proliferation, and collagen accumulation, the treatment with gene knockdown and SO_2_ donor in HPAECs showed that the deficiency of EC-derived SO_2_ promoted nuclear translocation of p50 in HPAECs and HPASMCs with significantly augmented PAEC inflammation, PASMC proliferation, and collagen remodelling. By contrast, the increase in EC-derived SO_2_ by AAT1 overexpression obviously inhibited hypoxia-induced activation of p50 in PAECs, thereby attenuating hypoxic PAEC inflammation, PASMC proliferation, and collagen remodelling *in vitro* and *in vivo*. More interestingly, the treatment with Andro, a p50 inhibitor, markedly blocked nuclear translocation of p50, and the subsequent PAEC inflammation, PASMC proliferation, and collagen accumulation induced by the decrease in EC-derived SO_2_ in HPAECs and HPASMCs. Therefore, these findings clarified that EC-derived SO_2_ inhibited p50 activation to control the inflammation of PAECs, and PASMC proliferation, and collagen accumulation.

The mechanism by which SO_2_ inhibits p50 activation has not yet been elucidated. The DNA-binding activity of p50 is regulated by the redox reaction, and NO nitrosylates the cysteine sulfhydryl groups of p50 and significantly inhibits its DNA-binding activity [40, 41]. Intriguingly, SO_2_ is also implicated in oxidative and reductive modification of proteins, such as AAT1 and p65, to modulate protein function in association with the cysteine sulfhydryl groups [42, 43]. In addition, previous studies also indicated that TGF-*β*/Smad, Raf-1/MEK-1/Erk/MAPK, AE2, and Dkk1/Wnt signalling pathways were implicated in the regulation of SO_2_ on PASMC proliferation, hypertrophy, and collagen deposition [35, 44–49]. In the future, further investigating the mechanism by which SO_2_ regulates p50 and revealing the potential clinical value of application of SO_2_ gas for the treatment of hypoxic PH are necessary.

## 5. Conclusions

The obtained data elucidated that endogenous SO_2_ in ECs exerted autocrine and paracrine effects to control PAEC inflammation, and PASMC proliferation, hypertrophy and collagen remodelling to inhibit PVR induced by hypoxia. The findings revealed a novel PAEC-PASMC communication mechanism in the endogenous control of hypoxic PVR by EC-derived SO_2_. Moreover, this study identified a potential target for the treatment of hypoxic PVR in aging-related cardiopulmonary diseases.

## Data Availability

All data needed to evaluate the conclusions in the paper are present in the paper and/or Supplementary Materials. Additional data related to this paper may be requested from the authors.

## Supplementary Materials

Supplementary 1 Figure S1: Detection of mouse genotype. The mice numbers are 1-9. Numbers 1, 3, and 7 represent wild-type (WT) mice. Numbers 2, 4-6, and 8-9 represent endothelial cell-specific AAT1 transgenic (EC-AAT1-Tg) mice.

Supplementary 2 Figure S2: Increased EC-derived SO_2_ ameliorates hypoxia-induced PAEC inflammation *in vivo* and *in vitro*. (a) Immunofluorescence *in situ* detection of MCP-1 protein expression in mouse PAECs. Red fluorescence represents MCP-1 protein and green fluorescence represents endothelial cell marker CD31. (b) *In situ* detection of macrophage infiltration around the mouse pulmonary arteries by immunofluorescence. Green fluorescence represents mouse macrophage marker F4/80, and red fluorescence represents vascular smooth muscle marker *α*-SMA. (c, d) Western blot analysis (*n* = 9) and immunofluorescence methods were used to detect the expression level of ICAM-1 protein in HPAECs. Yellow fluorescence represents ICAM-1. (e) Western blot analysis was used to detect the expression level of MCP-1 protein in HPAECs (*n* = 9). (f) Fluorescence method was performed to detect the adhesion of THP-1 cells and HPAECs. Red Dil staining marks THP-1 cells and DAPI color marks the nuclei of HPAECs. The data were expressed as mean ± SEM, ^∗^*p* < 0.05, scale: 20 *μ*m.

Supplementary 3 Figure S3: EC-derived SO_2_ deficiency stimulated HPASMC hypertrophy *in vitro.* (a) The expression level of *α*-SMA protein, a marker of smooth muscle cell hypertrophy, in HPASMCs cocultured with HPAECs was detected by Western blot method (*n* = 9). (b) The expression level of *α*-SMA protein in HPASMCs cocultured with HPAECs *in situ* was observed by immunofluorescence method. The red fluorescence represents *α*-SMA protein and blue DAPI color marks the nuclei of HPASMCs. The data were expressed as mean ± SEM, ^∗^*p* < 0.05, scale bar: 20 *μ*m.

Supplementary 4 Figure S4: AAT1 overexpression inhibits hypoxia-induced activation of p50 in PAECs. (a) Western blot analysis was used to detect the phosphorylation level of p50 protein in HPAECs (*n* = 9). (b) The distribution of p50 protein in HPAECs was detected by immunofluorescence *in situ*. Green fluorescence represents p50 protein. (c) Active motif-ELISA was performed to detect the DNA-binding activity of p50 in HPAECs (*n* = 9). (d) Immunofluorescence was used to *in situ* detect p-p50 protein expression in mouse PAECs, green fluorescence represents p-p50 protein. The data were expressed as mean ± SEM, ^∗^*p* < 0.05, scale: 20 *μ*m.
