# Supplementary figures and images for: Endothelial Cell-Derived SO2 Controls Endothelial Cell Inflammation, Smooth Muscle Cell Proliferation, and Collagen Synthesis to Inhibit Hypoxic Pulmonary Vascular Remodelling
Source: Oxid Med Cell Longev. 2021 Apr 17;2021:5577634. doi: 10.1155/2021/5577634 (PMC8068783; doi:10.1155/2021/5577634)

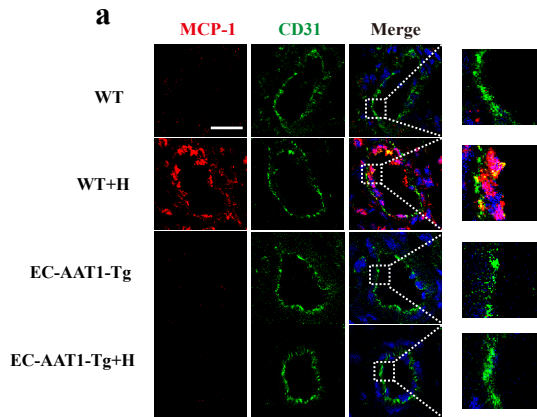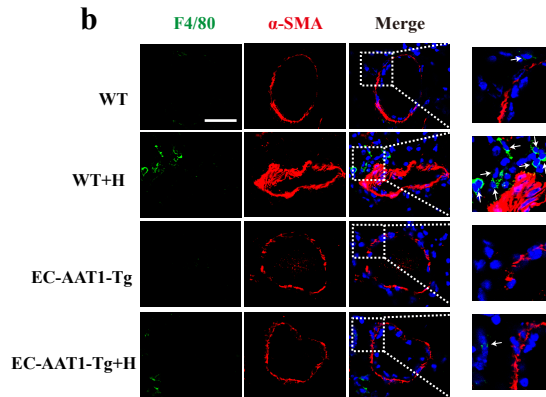

Supplement: Supplementary 2 — Figure S2: Increased EC-derived SO2 ameliorates hypoxia-induced PAEC inflammation in vivo and in vitro. (a) Immunofluorescence in situ detection of MCP-1 protein expression in mouse PAECs. Red fluorescence represents MCP-1 protein and green fluorescence represents endothelial cell marker CD31. (b) In situ detection of macrophage infiltration around the mouse pulmonary arteries by immunofluorescence. Green fluorescence represents mouse macrophage marker F4/80, and red fluorescence represents vascular smooth muscle marker α-SMA. (c, d) Western blot analysis (n = 9) and immunofluorescence methods were used to detect the expression level of ICAM-1 protein in HPAECs. Yellow fluorescence represents ICAM-1. (e) Western blot analysis was used to detect the expression level of MCP-1 protein in HPAECs (n = 9). (f) Fluorescence method was performed to detect the adhesion of THP-1 cells and HPAECs. Red Dil staining marks THP-1 cells and DAPI color marks the nuclei of HPAECs. The data were expressed as mean ± SEM, ∗p < 0.05, scale: 20 μm. [file 5577634.f2.zip › Fig. S2a-S2b.pdf]

**c**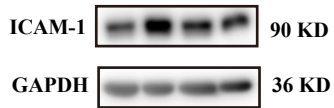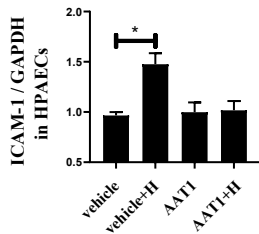**d**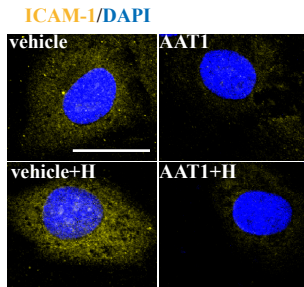

Supplement: Supplementary 2 — Figure S2: Increased EC-derived SO2 ameliorates hypoxia-induced PAEC inflammation in vivo and in vitro. (a) Immunofluorescence in situ detection of MCP-1 protein expression in mouse PAECs. Red fluorescence represents MCP-1 protein and green fluorescence represents endothelial cell marker CD31. (b) In situ detection of macrophage infiltration around the mouse pulmonary arteries by immunofluorescence. Green fluorescence represents mouse macrophage marker F4/80, and red fluorescence represents vascular smooth muscle marker α-SMA. (c, d) Western blot analysis (n = 9) and immunofluorescence methods were used to detect the expression level of ICAM-1 protein in HPAECs. Yellow fluorescence represents ICAM-1. (e) Western blot analysis was used to detect the expression level of MCP-1 protein in HPAECs (n = 9). (f) Fluorescence method was performed to detect the adhesion of THP-1 cells and HPAECs. Red Dil staining marks THP-1 cells and DAPI color marks the nuclei of HPAECs. The data were expressed as mean ± SEM, ∗p < 0.05, scale: 20 μm. [file 5577634.f2.zip › Fig. S2c-S2d.pdf]

**e**

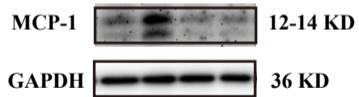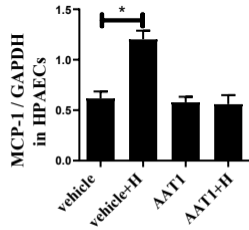

**f**

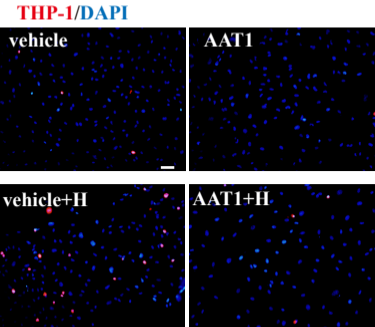

Supplement: Supplementary 2 — Figure S2: Increased EC-derived SO2 ameliorates hypoxia-induced PAEC inflammation in vivo and in vitro. (a) Immunofluorescence in situ detection of MCP-1 protein expression in mouse PAECs. Red fluorescence represents MCP-1 protein and green fluorescence represents endothelial cell marker CD31. (b) In situ detection of macrophage infiltration around the mouse pulmonary arteries by immunofluorescence. Green fluorescence represents mouse macrophage marker F4/80, and red fluorescence represents vascular smooth muscle marker α-SMA. (c, d) Western blot analysis (n = 9) and immunofluorescence methods were used to detect the expression level of ICAM-1 protein in HPAECs. Yellow fluorescence represents ICAM-1. (e) Western blot analysis was used to detect the expression level of MCP-1 protein in HPAECs (n = 9). (f) Fluorescence method was performed to detect the adhesion of THP-1 cells and HPAECs. Red Dil staining marks THP-1 cells and DAPI color marks the nuclei of HPAECs. The data were expressed as mean ± SEM, ∗p < 0.05, scale: 20 μm. [file 5577634.f2.zip › Fig. S2e-S2f.pdf]

**a**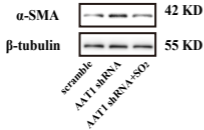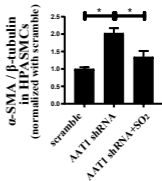**b**

scramble

AAT1 shRNA

AAT1 shRNA+SO<sub>2</sub> $\alpha$ -SMA

DAPI

HPASMCs

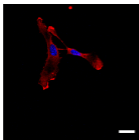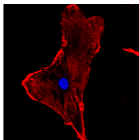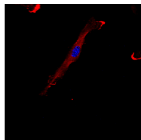

Supplement: Supplementary 3 — Figure S3: EC-derived SO2 deficiency stimulated HPASMC hypertrophy in vitro. (a) The expression level of α-SMA protein, a marker of smooth muscle cell hypertrophy, in HPASMCs cocultured with HPAECs was detected by Western blot method (n = 9). (b) The expression level of α-SMA protein in HPASMCs cocultured with HPAECs in situ was observed by immunofluorescence method. The red fluorescence represents α-SMA protein and blue DAPI color marks the nuclei of HPASMCs. The data were expressed as mean ± SEM, ∗p < 0.05, scale bar: 20 μm. [file 5577634.f3.pdf]

**a**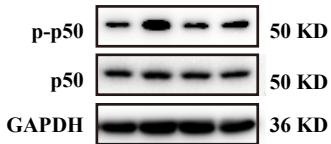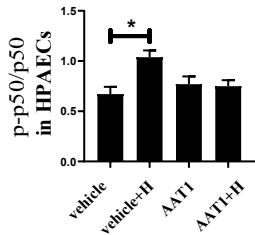**b**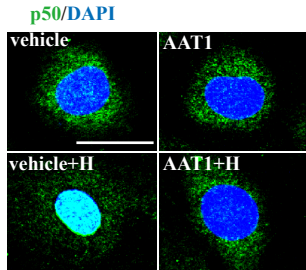

Supplement: Supplementary 4 — Figure S4: AAT1 overexpression inhibits hypoxia-induced activation of p50 in PAECs. (a) Western blot analysis was used to detect the phosphorylation level of p50 protein in HPAECs (n = 9). (b) The distribution of p50 protein in HPAECs was detected by immunofluorescence in situ. Green fluorescence represents p50 protein. (c) Active motif-ELISA was performed to detect the DNA-binding activity of p50 in HPAECs (n = 9). (d) Immunofluorescence was used to in situ detect p-p50 protein expression in mouse PAECs, green fluorescence represents p-p50 protein. The data were expressed as mean ± SEM, ∗p < 0.05, scale: 20 μm. [file 5577634.f4.zip › Fig. S4a-S4b.pdf]

**c**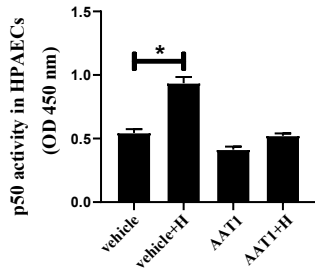**d**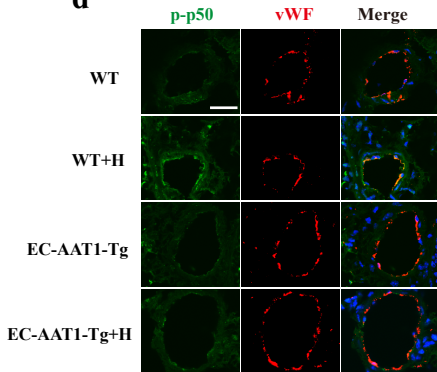

Supplement: Supplementary 4 — Figure S4: AAT1 overexpression inhibits hypoxia-induced activation of p50 in PAECs. (a) Western blot analysis was used to detect the phosphorylation level of p50 protein in HPAECs (n = 9). (b) The distribution of p50 protein in HPAECs was detected by immunofluorescence in situ. Green fluorescence represents p50 protein. (c) Active motif-ELISA was performed to detect the DNA-binding activity of p50 in HPAECs (n = 9). (d) Immunofluorescence was used to in situ detect p-p50 protein expression in mouse PAECs, green fluorescence represents p-p50 protein. The data were expressed as mean ± SEM, ∗p < 0.05, scale: 20 μm. [file 5577634.f4.zip › Fig. S4c-S4d.pdf]
